# Physiotherapeutic Management of Adolescent Idiopathic Scoliosis: A Focused Review of the Schroth Method

**DOI:** 10.3390/jcm15031266

**Published:** 2026-02-05

**Authors:** Alexandru Herdea, Alexandru Ionuț Ciobanu, Alexandru Ulici

**Affiliations:** 1 11th Department of Pediatric Orthopedics, Carol Davila University of Medicine and Pharmacy, Bd. Eroii Sanitari Nr. 8, 050474 Bucharest, Romania; alexandru.herdea@umfcd.ro (A.H.); alexandru.ulici@umfcd.ro (A.U.); 2 Pediatric Orthopedics Department, Grigore Alexandrescu Children’s Emergency Hospital, 011743 Bucharest, Romania

**Keywords:** adolescent idiopathic scoliosis, Schroth Method, physiotherapeutic scoliosis-specific exercises, three-dimensional correction, conservative management, postural neuromuscular control

## Abstract

Adolescent idiopathic scoliosis (AIS) is a three-dimensional deformity of the growing spine, frequently associated with functional impairment, altered trunk biomechanics, compromised respiratory performance, and psychosocial burden. The risk of curve progression increases during periods of rapid growth, highlighting the need for effective conservative interventions targeting both structural and neuromuscular components of the deformity. This review synthesizes evidence published between 2005 and 2025 on the effects of Schroth-based physiotherapeutic scoliosis-specific exercises in adolescents aged 10–18 years with idiopathic scoliosis and Risser stages 0–5. Studies applying Schroth therapy exclusively or predominantly, either as a stand-alone intervention or combined with bracing, were included, while non-idiopathic scoliosis and mixed PSSE protocols were excluded. A total of 17 studies meeting predefined eligibility criteria were included. Across randomized controlled trials, controlled cohort studies, and longitudinal case series, Schroth interventions were associated with attenuation of Cobb angle progression, improvements in three-dimensional trunk symmetry, postural control, respiratory mechanics, and health-related quality of life. Combined Schroth and brace therapy generally demonstrated superior outcomes compared with bracing alone. Despite these findings, heterogeneity in intervention protocols and outcome measures limits direct comparability across studies. Overall, current evidence supports the Schroth Method as a relevant conservative strategy for AIS, particularly when initiated early and delivered through individualized three-dimensional correction. Further high-quality multicenter studies with standardized protocols are required to strengthen the long-term evidence base.

## 1. Introduction

Adolescent idiopathic scoliosis (AIS) is a complex, three-dimensional spinal deformity emerging during the critical window of adolescent growth, characterized not merely by a lateral curvature exceeding 10 degrees, but by a coupled deformity involving vertebral rotation, rib cage distortion, alteration of sagittal alignment, and asymmetric growth kinetics [1,2,3,4]. Affecting approximately 2–3% of otherwise healthy adolescents, AIS unfolds within a biomechanical and neurodevelopmental environment marked by rapid longitudinal bone growth, evolving neuromuscular control, and dynamic sensorimotor integration. These factors together create a uniquely vulnerable context in which minor perturbations can propagate nonlinearly, enabling relatively small initial asymmetries to trigger significant structural progression [1,2,3,5].

Although the etiology of AIS remains elusive, contemporary high-quality research increasingly supports a multifactorial systems-based model, integrating genetic determinants (including polygenic susceptibility loci), asymmetric vertebral growth governed by the Hueter–Volkmann law, impaired postural and proprioceptive control, neuromuscular dyscoordination of paraspinal stabilizers, vestibular dysregulation, and perturbations in central nervous system processing [1,5]. These interacting mechanisms suggest that AIS arises not from a single anatomical defect, but from a broader disturbance in how the developing spine responds to biomechanical loading, growth modulation forces, and neurosensory feedback [1,2,3,5].

Beyond radiographic metrics, AIS carries a substantial functional and psychosocial burden [1,2,3,5]. The triad of trunk asymmetry, thoracic rotation, and postural imbalance alters biomechanical efficiency, compromises respiratory mechanics, and distorts body schema. Consequently, adolescents frequently experience reduced self-esteem, altered self-perception, social withdrawal, and diminished involvement in physical activities [2]. These psychosocial effects are increasingly recognized not as secondary concerns but as primary clinical outcomes that influence adherence, long-term prognosis, and quality of life [2].

Conservative management remains fundamental for skeletally immature patients, especially those at risk of progression. While observation and rigid bracing have long constituted the backbone of treatment decisions, the last two decades have marked a paradigm shift toward active, neuromuscularly driven interventions. Physiotherapeutic scoliosis-specific exercises (PSSE) aim to modulate the pathomechanics of AIS by targeting asymmetrical muscle activation, correcting faulty postural strategies, and influencing vertebral growth orientation through repeated, task-specific sensorimotor stimuli [1,2,3,5].

Among physiotherapeutic scoliosis-specific exercise (PSSE) approaches, the Schroth Method represents one of the most extensively investigated and clinically applied schools within the broader PSSE framework, with growing support from randomized controlled trials and international consensus-based recommendations [1,5,6].

The method directly addresses the biomechanical coupling inherent to AIS: lateral deviation, vertebral rotation, and sagittal imbalance [1,2,3,5]. Through carefully structured neuromuscular activation patterns, Schroth exercises aim to modify trunk morphology, normalize postural strategies, reorganize proprioceptive mapping, and create sustained corrective forces with potential influence on growth modulation through sustained corrective loading [7,8,9,10,11,12,13,14,15,16,17].

Given the exponential increase in high-quality clinical trials, imaging-based studies, biomechanics research, and longitudinal follow-up analyses published from 2005 to 2025, a rigorous synthesis focusing exclusively on Schroth interventions in AIS is warranted [1,2,3,5]. Previous reviews have often merged multiple PSSE schools, limiting the ability to isolate Schroth-specific effects. To advance the field, a dedicated examination of Schroth’s methodological impact on structural deformity, trunk kinetics, respiratory function, neuromuscular outcomes, and psychosocial indices is required [1,2,3,5]. Despite substantial clinical interest, the existing literature remains methodologically heterogeneous, with many reviews aggregating multiple PSSE schools and thereby obscuring Schroth-specific effects. This gap underscores the need for a focused synthesis capable of isolating the independent therapeutic impact of Schroth-based interventions [1,5].

**Figure 1:**
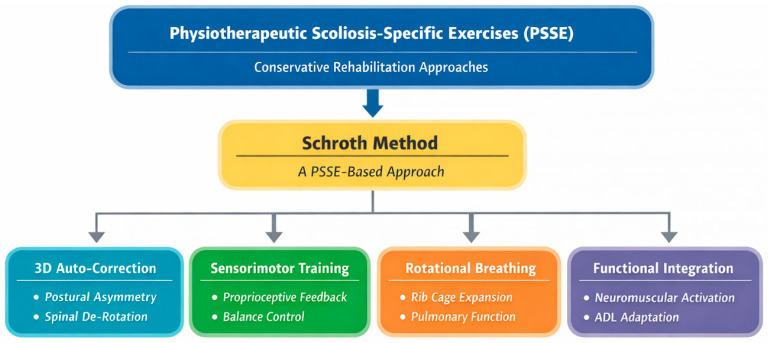
Conceptual mechanistic model of Schroth Method in adolescent idiopathic scoliosis. Abbreviations: ADL—activities of daily living. Physiotherapeutic scoliosis-specific exercises (PSSE) represent an umbrella framework encompassing multiple scoliosis-specific rehabilitation schools. The Schroth Method is illustrated here as a distinct PSSE-based approach characterized by three-dimensional auto-correction, rotational angular breathing, and functional integration, which modulate asymmetric loading, vertebral growth kinetics, rib cage deformity, proprioceptive dysregulation, and neuromuscular imbalance. These mechanisms collectively contribute to improvements in structural alignment, trunk biomechanics, respiratory function, and patient-reported outcomes, with enhanced effects when combined with rigid bracing.

The present review integrates two decades of research to critically evaluate the therapeutic influence of Schroth-based interventions in adolescents with idiopathic scoliosis. By excluding non-idiopathic etiologies and other PSSE approaches, the review offers a refined and theory-grounded contribution to understanding the role of Schroth therapy within contemporary conservative scoliosis management [6,7,8,9,10,11,12,13,14,15,16,17,18,19,20,21,22]. A conceptual mechanistic model summarizing the theoretical pathways through which Schroth therapy exerts its effects in AIS is presented in Figure 1.

## 2. Materials and Methods

This systematic review was conducted in accordance with the PRISMA 2020 guidelines and was retrospectively registered on the Open Science Framework (OSF), Registration ID: https://osf.io/kz9mu.

The protocol for this review, including eligibility criteria, search strategy, study selection process, and planned synthesis methods, was registered retrospectively on OSF (https://osf.io/kz9mu). No deviations from the protocol occurred. This review focuses exclusively on the effects of Schroth-based physiotherapeutic scoliosis-specific exercises (PSSE) in adolescents with idiopathic scoliosis (AIS).

This review was designed as a structured narrative review, not as a formal systematic review or meta-analysis. Elements of the PRISMA framework were selectively applied to improve transparency in reporting the search strategy and study selection process. Specifically, we used a PRISMA-style flow diagram to document identification, screening, eligibility assessment, and final inclusion of studies. Other PRISMA components that are strictly required for systematic reviews (e.g., protocol registration, exhaustive database coverage, quantitative synthesis) were not applied because the objective of the present work was qualitative synthesis rather than statistical pooling.

### 2.1. Eligibility Criteria

Studies were included if they met the following criteria:

(1) participants aged 10–18 years with adolescent idiopathic scoliosis (AIS), Risser stage 0–5;
(2) Schroth Method used as the predominant or exclusive physiotherapeutic intervention, with or without rigid bracing;
(3) reporting outcomes such as Cobb angle, angle of trunk rotation (ATR), trunk morphology, respiratory function, neuromuscular control, or quality of life;
(4) randomized controlled trials, controlled clinical trials, or prospective/retrospective cohort studies.

Exclusion criteria: non-idiopathic scoliosis types, mixed PSSE protocols without isolated Schroth results, surgical or pharmacologic studies, abstracts, letters, reviews, editorials, and studies lacking extractable data.

### 2.2. Information Sources

A systematic literature search was conducted in PubMed, ScienceDirect, and Google Scholar for studies published between January 2005 and December 2025. Reference lists of included studies and relevant clinical texts were also screened.

### 2.3. Search Strategy

The primary PubMed search strategy was: (“adolescent idiopathic scoliosis” OR “AIS”) AND (“Schroth method” OR “Schroth exercises” OR “physiotherapeutic scoliosis-specific exercises” OR “PSSE”).

Filters applied: English language, human subjects, adolescent population.

The strategy was adapted appropriately for ScienceDirect and Google Scholar.

Study selection and data extraction were performed by two independent reviewers (A.H. and A.I.C.). Titles and abstracts were screened first, followed by full-text evaluation of potentially eligible studies. Minor disagreements that arose during screening were resolved through discussion, by weighing arguments for inclusion or exclusion in each case. Because consensus was always reached through dialogue, formal arbitration by the senior reviewer (A.U.) was not required. Data extraction included study design, population characteristics, intervention details, outcomes, and main results. Risk of bias was assessed independently by two reviewers (A.H. and A.I.C.) using domain-based criteria adapted from the Cochrane risk-of-bias recommendations. Minor discrepancies were resolved through discussion and consensus between the two reviewers; no external arbitration was required. The results of the risk-of-bias assessment are summarized in the following section.

### 2.4. Study Selection

Two reviewers independently screened titles and abstracts. Full-text screening was performed for all potentially relevant articles. Disagreements were resolved through discussion and consensus. The PRISMA 2020 flow diagram presents the selection process.

### 2.5. Data Collection Process

Data extraction was conducted independently by two reviewers using a structured extraction sheet. Extracted data included: study design, sample size, participant characteristics, curve magnitude and type, skeletal maturity, intervention dosage and supervision, bracing status, and all clinical/radiographic outcomes. Data were cross-verified for accuracy.

### 2.6. Data Items

Primary outcomes: Cobb angle, angle of trunk rotation, and three-dimensional trunk morphology.

Secondary outcomes: respiratory parameters (FVC, FEV1), postural indices, neuromuscular function, and quality of life measured via SRS-22.

### 2.7. Risk of Bias Assessment

The methodological quality and risk of bias of the included studies were independently assessed by two reviewers. For randomized controlled trials, the Cochrane Risk of Bias 2.0 tool was applied, evaluating domains including randomization process, allocation concealment, blinding, completeness of outcome data, and selective reporting. For non-randomized and observational studies, the Newcastle–Ottawa Scale (NOS) and MINORS criteria were used, as appropriate. Discrepancies between reviewers were resolved through discussion with a third senior author until consensus was reached.

The detailed results of the quality assessment for each study are summarized Below.

### 2.8. Effect Measures

For continuous outcomes, mean differences (MD), percent changes, and confidence intervals were extracted where available. Substantial heterogeneity in outcome measures and intervention protocols prevented meta-analysis.

### 2.9. Synthesis Methods

A qualitative synthesis approach was used. Studies were categorized into two groups: (1) Schroth-only interventions; and (2) Schroth combined with bracing. Heterogeneity in treatment dosage, supervision frequency, and outcome measures precluded quantitative synthesis.

### 2.10. Reporting Bias and Certainty Assessment

Selective outcome reporting and missing data were evaluated qualitatively. Certainty of evidence for primary and secondary outcomes was assessed using a simplified GRADE framework.

#### PRISMA-Guided Study Selection

Study identification, screening, eligibility assessment, and inclusion followed the PRISMA 2020 framework. A total of 582 records were identified across databases (PubMed *n* = 109; ScienceDirect *n* = 91; Google Scholar *n* = 382). After removal of 110 duplicates, 472 records underwent title and abstract screening, of which 382 were excluded for not meeting inclusion criteria. Ninety full-text articles were assessed for eligibility, and 72 were excluded for reasons including non-idiopathic scoliosis (*n* = 12), mixed PSSE protocols (*n* = 28), lack of Schroth-specific outcomes (*n* = 18), insufficient data (*n* = 9), and non-physiotherapy interventions (*n* = 6). A total of 17 studies met all predefined criteria and were included in the final qualitative synthesis. The complete selection process is presented in the PRISMA flow diagram (Figure 1).

Although this manuscript represents a structured narrative review rather than a full systematic review or meta-analysis, key elements of the PRISMA 2020 guideline were intentionally incorporated to enhance methodological transparency and reproducibility.

The following PRISMA components were applied:

– clear definition of inclusion and exclusion criteria
– specification of databases searched and full search strategy
– reporting of the time frame of the literature search
– structured process of study identification, screening, eligibility assessment, and inclusion
– use of a PRISMA-style flow diagram to document the selection process
– qualitative synthesis of the included studies

Several elements were adapted to fit a narrative design:

– data were synthesized descriptively rather than quantitatively
– heterogeneity between protocols precluded meta-analysis
– risk-of-bias evaluation was performed using domain-based qualitative criteria rather than pooled scoring systems

The following elements were not applied, as they are specific to full systematic reviews and meta-analyses:

– protocol registration (e.g., PROSPERO)
– quantitative effect-size estimation
– meta-analytic pooling
– assessment of publication bias through funnel-plot analysis

Therefore, PRISMA principles were used to structure transparency of reporting, while methodological flexibility consistent with a narrative review was maintained.

**Figure 2:**
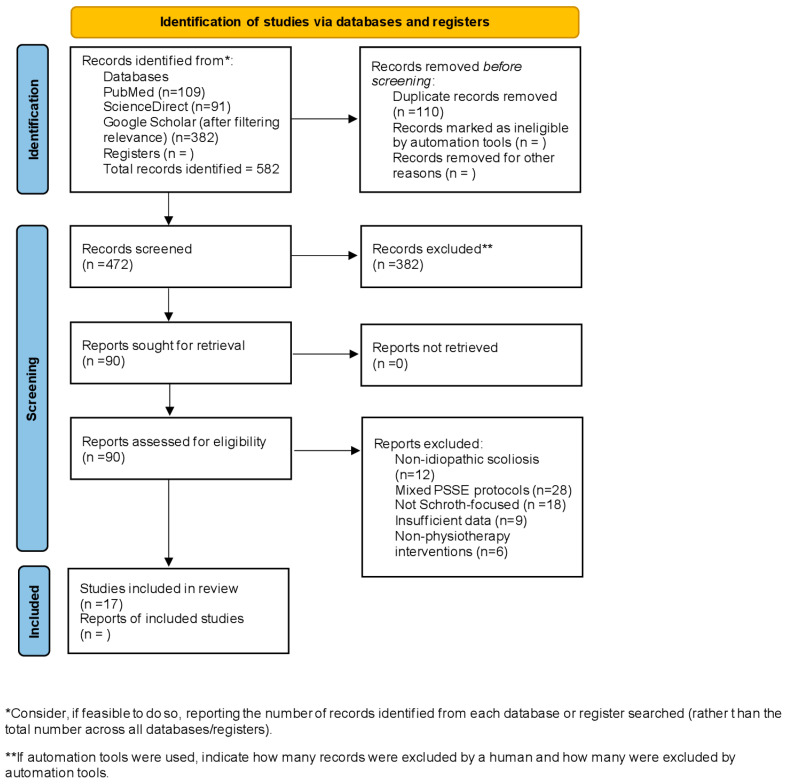
PRISMA 2020 flow diagram for study selection [23].

The diagram (Figure 2) illustrates the identification, screening, eligibility assessment, and final inclusion of studies evaluating Schroth-based physiotherapeutic interventions for AIS. A total of 17 studies met inclusion criteria. Adapted from Supplementary Materials PRISMA 2020 Statement (Page et al., BMJ 2021 [23]).

Risk of bias was generally low to moderate among the included studies, with most randomized controlled trials demonstrating adequate methodological rigor. A detailed assessment is presented in the Discussion Section Certainty of evidence for primary and secondary outcomes was evaluated using a simplified GRADE approach and is summarized in Discussion Section.

Risk of bias for randomized controlled trials (RCTs) was assessed using the RoB 2.0 tool, and non-randomized studies were evaluated using the ROBINS-I tool. Overall, most studies demonstrated low to moderate risk of bias.

## 3. Results

The studies included in this review consistently demonstrate that Schroth-based physiotherapy exerts measurable and clinically meaningful effects on the structural, functional, respiratory, and psychosocial dimensions of adolescent idiopathic scoliosis. Across the examined literature, the most robust improvements occurred in adolescents with mild to moderate curves. Importantly, greater skeletal immaturity (Risser 0–2), which is associated with a higher risk of curve progression, underscores the necessity of initiating conservative treatment as early as possible. Early intervention may enhance the capacity of neuromuscular training to counteract asymmetric loading and mitigate progression-related forces [7,8,9,10,11,13,15,16,17].

Although the magnitude of improvement varied across studies, most investigations reported clinically relevant stabilization or mild regression of curvature, particularly in thoracic and thoracolumbar patterns. Variability in effect size appeared to correlate with intervention dosage, supervision quality, and baseline skeletal maturity [8,11,13,15,17].

A detailed overview of the included trials is presented in Table 1 (Schroth-only interventions) and Table 2 (Schroth combined with bracing). These tables summarize sample characteristics, curve severity, skeletal maturity, intervention protocols, follow-up duration, and the main reported outcomes.

Randomized and non-randomized controlled trials comparing Schroth interventions with standard care—typically observation, generic physiotherapy, or non-curve-specific exercise programs—reported significant reductions in Cobb angle progression, with several studies documenting modest but statistically relevant curve regression [8,11,13]. Protocols spanning 6 to 12 months, combining structured supervised sessions with high-frequency home programs, consistently yielded the most favorable outcomes [8,9,10,11,13]. Improvements were not limited to radiographic parameters; multiple studies reported enhanced three-dimensional trunk morphology, including reductions in angle of trunk rotation, normalization of shoulder and pelvic balance, recalibration of sagittal alignment, and improved dynamic postural strategies [6,8,9,10,11,12,15,19].

When Schroth therapy was integrated with rigid bracing, outcomes were uniformly superior to those achieved through bracing alone. Adolescents receiving combined therapy demonstrated more symmetrical loading patterns, better stabilization within the orthosis, and improved brace tolerance—a factor strongly correlated with long-term adherence [5,6,18,19,20,21,22]. Radiographic follow-up often revealed lower progression rates and, in selected cases, mild curve improvement, suggesting that Schroth-specific neuromuscular corrections may potentiate the biomechanical effects of bracing during critical growth periods [6,18,19,21,22]. A small subset of studies reported neutral or modest effects [16,17], underscoring the influence of protocol adherence and therapist expertise on treatment outcomes.

Respiratory and thoracic parameters also showed consistent improvement. Schroth’s rotational angular breathing facilitated increased chest wall mobility, enhanced thoracic expansion on the concave side, and improved ventilation asymmetry, which are particularly relevant given the restrictive ventilatory tendencies associated with thoracic curve patterns [12,13,14,20,21]. Several studies documented measurable improvements in lung function indices, respiratory mechanics, and thoracic kinematics [7,8,14,21].

Psychosocial and patient-centered outcomes were equally notable. Across studies employing the SRS-22 and related quality-of-life instruments, participants demonstrated improvements in self-image, pain, function, and mental health domains. Enhanced self-perception of symmetry, increased confidence in physical activity, and overall improved body schema were repeatedly reported [6,8,10,11,13,15,19,22]. These findings underscore the relevance of Schroth therapy not only as a structural intervention but as a holistic rehabilitative method influencing psychological resilience and daily life performance.

## 4. Discussion

The findings of this twenty-year synthesis reinforce and extend the emerging consensus that Schroth-based physiotherapy represents a biomechanically plausible and clinically meaningful conservative intervention for adolescent idiopathic scoliosis (AIS). Unlike general exercise programs that fail to address the inherent three-dimensional coupling of the deformity, the Schroth Method specifically targets the pathological interactions between spinal alignment, rib cage morphology, neuromuscular activation, and sensorimotor integration [1,2,3,5]. By directing corrective forces along all three spatial planes, Schroth training seeks not only to mitigate curve progression but also to reorganize trunk biomechanics and recalibrate postural control strategies that are often disrupted in AIS [7,8,9,10,11,12,13,14,15,16,17].

Consistent with the findings of this review, most studies reported favorable structural, functional, and patient-reported outcomes following Schroth-based physiotherapy. However, substantial heterogeneity persists in exercise dosage, supervision intensity, progression algorithms, and outcome metrics. This variability limits direct comparability across studies and precludes robust meta-analytic synthesis. Despite these limitations, the convergent direction of outcomes suggests that Schroth-based physiotherapy represents a clinically meaningful and biomechanically plausible conservative intervention for adolescents with idiopathic scoliosis.

The characteristics of studies evaluating combined Schroth and brace interventions are presented in Table 3.

The core principles of the method—three-dimensional auto-correction, rotational angular breathing, and integration of corrected patterns into functional movement—align closely with current mechanobiological theories of scoliosis development [1,2,3,5]. Three-dimensional auto-correction directly influences the asymmetric loading patterns implicated in progressive deformity, while rotational angular breathing has the potential to modulate thoracic mechanics by actively expanding concave segments of the rib cage and improving ventilation asymmetry [12,13,14,20]. The functional integration component further reinforces corrected trunk alignment through repeated motor learning cycles, supporting durable neuromuscular adaptations and potentially influencing growth modulation through sustained postural forces [7,8,9,10,11,12,13,14,15,16,17].

From a mechanobiological perspective, it has been hypothesized that repeated application of asymmetric corrective loading may contribute to growth modulation according to the Hueter–Volkmann principle. However, the growth pattern in AIS is complex and regionally differentiated, with evidence suggesting relatively increased anterior vertebral growth in many curves. This raises an important conceptual question as to why exercises that frequently incorporate elements of axial elongation and extension can nevertheless yield favorable clinical outcomes. A plausible explanation is that Schroth exercises do not simply act through sagittal-plane extension, but rather through three-dimensional autocorrection, active derotation, improved neuromuscular control, and redistribution of asymmetrical loading across the spine and rib cage. Future studies integrating three-dimensional imaging and motion analysis are required to further clarify these mechanisms.

The evidence synthesized here indicates that Schroth interventions are most effective when implemented early, particularly in skeletally immature patients (Risser 0–2) who exhibit the greatest susceptibility to progression [7,8,9,10,11]. Early neuromuscular re-education may disrupt the feed-forward mechanisms that perpetuate asymmetric loading and may improve the spine’s capacity to tolerate and redistribute mechanical stresses during peak growth velocity [8,9,10,11]. When paired with rigid bracing, Schroth training appears to augment the biomechanical efficacy of orthotic correction. This synergy likely arises from enhanced proprioceptive awareness, improved trunk stabilization within the brace, and reduced compensatory postures that undermine the intended corrective forces of the orthosis [6,18,19,20,21,22].

Notably, a minority of studies documented limited or non-significant radiographic changes [16,17]. These discrepancies may reflect differences in intervention fidelity, therapist certification level, brace compliance, or inadequate exercise dosage. Such variability highlights the need for stricter reporting standards and the development of validated fidelity metrics to ensure consistent application of Schroth principles across clinical settings.

Nevertheless, the substantial heterogeneity observed across studies underscores persistent methodological challenges. Protocols vary widely in exercise dosage, supervision intensity, duration of intervention, and adherence monitoring, making direct comparison difficult and limiting the ability to derive standardized clinical guidelines [4]. Moreover, the quality, frequency, and progression of supervised sessions—variables often insufficiently reported—may exert a decisive influence on clinical outcomes. Therapist expertise, adherence strategies, and individualized progression models represent critical components of treatment efficacy and must be more rigorously accounted for in future research.

Another limitation arises from variability in outcome measures. While Cobb angle remains the dominant radiographic metric, AIS is fundamentally a three-dimensional disorder involving rotational, sagittal, and functional components that are not always fully captured by two-dimensional imaging. Future studies should incorporate more sophisticated assessments, including surface topography, three-dimensional imaging, rib cage kinematics, neuromuscular activation patterns, and validated patient-reported outcomes [4]. Understanding how Schroth therapy influences these multidimensional parameters will be essential for clarifying its therapeutic mechanisms.

Advances in digital health technologies offer promising opportunities to improve treatment fidelity and data granularity. Wearable sensors, mobile-based training platforms, and remote adherence monitoring systems may help quantify real-time posture, movement quality, and exercise precision—metrics that have historically been difficult to evaluate [4]. These innovations could support more ecologically valid assessments of daily postural strategies and enable more personalized, adaptive training models.

Quality-of-life assessment scores should also be applied to patients in the preoperative phase, particularly after completing physiotherapy programs or undergoing brace treatment, as these interventions can significantly influence patients’ daily functioning and overall well-being. Such an approach would provide a more comprehensive understanding of treatment impact, similar to the pre- and postoperative evaluations typically performed in surgical cohorts. This perspective aligns with previous findings showing meaningful changes in quality of life among children and adolescents with idiopathic scoliosis following surgical management [24].

Overall, the accumulated evidence suggests that Schroth-based physiotherapy has the potential to influence both structural and functional characteristics of AIS, particularly when applied within a high-quality, individualized, and intensively supervised framework [6,7,8,9,10,11,12,13,14,15,16,17,18,19,20,21,22]. However, to elevate this field to the standards expected by the international spine community, future research must prioritize methodological standardization, mechanistic exploration, and longitudinal tracking into skeletal maturity. Such advancements are essential for defining minimal effective dosage, optimizing treatment algorithms, and clarifying the long-term impact of Schroth interventions on spinal health and quality of life.

### 4.1. Limitations

This review is subject to several limitations that warrant consideration. First, the heterogeneity across included studies—particularly regarding intervention dosage, duration of supervised sessions, therapist expertise, and adherence monitoring—limits the ability to derive standardized treatment parameters or conduct meta-analytic synthesis [4]. Second, the predominance of small-sample cohorts and single-center trials restricts generalizability, as results may be influenced by local clinical expertise and variability in protocol implementation.

Third, most studies relied primarily on two-dimensional radiographic measures such as Cobb angle, which insufficiently capture the three-dimensional complexity of scoliosis deformity. Few investigations incorporated advanced imaging, surface topography, or neuromuscular assessments that could elucidate mechanistic pathways of correction [4]. Lastly, long-term follow-up into late skeletal maturity and adulthood remains limited, constraining our understanding of the durability of Schroth-induced structural and functional changes.

#### 4.1.1. Risk of Bias Assessment

A risk of bias assessment was conducted for all included studies. The majority of randomized controlled trials presented low risk of bias, whereas several non-randomized designs showed some concerns. The detailed results are presented in Table 4.

#### 4.1.2. Future Directions

Future research in Schroth-based physiotherapy should prioritize standardized, multicenter randomized controlled trials employing unified intervention frameworks with clearly defined dosage, progression, and adherence criteria [4]. High-resolution three-dimensional imaging, wearable sensor systems, and objective neuromuscular assessments should be integrated to better characterize corrective mechanisms and quantify trunk biomechanics in real time.

Studies should also explore the minimal effective dose of supervised therapy, digital and hybrid rehabilitation models, and the differential effectiveness of Schroth therapy across distinct curve phenotypes, growth stages, and risk stratification profiles. Longitudinal follow-up into adulthood is essential to determine whether early neuromuscular correction influences long-term spinal health, pain trajectories, respiratory outcomes, and overall quality of life.

#### 4.1.3. Clinical Implications

The findings of this review suggest that Schroth-based physiotherapy may represent an important component of conservative management in adolescents with idiopathic scoliosis, particularly in patients at risk of curve progression. When applied in combination with bracing, Schroth exercises appear to enhance brace tolerance, improve postural control, and support adherence to treatment. Nevertheless, given the heterogeneity of available protocols and the limited number of high-quality long-term studies, these implications should be interpreted with caution. Clinical decision-making should remain individualized, and Schroth therapy should be considered as part of a comprehensive multidisciplinary approach rather than a stand-alone solution.

## 5. Conclusions

The accumulated evidence from the past two decades positions the Schroth Method as a robust, biomechanically grounded, and clinically effective component of conservative management for adolescent idiopathic scoliosis. When implemented early in skeletally immature patients and delivered through individualized, curve-specific, and adequately supervised protocols, Schroth-based interventions can meaningfully attenuate curve progression and enhance three-dimensional trunk alignment, respiratory mechanics, and patient-reported outcomes. The additive benefit observed when Schroth therapy is combined with rigid bracing underscores the value of integrated, multimodal, and patient-centered treatment strategies.

Despite these promising results, the field continues to be limited by substantial variability in intervention dosage, training progression, and outcome metrics. High-quality multicenter trials using standardized protocols and long-term follow-up into skeletal maturity are essential to refine therapeutic algorithms, identify minimal effective dosing parameters, and clarify the durability of treatment effects. Until such evidence emerges, the Schroth Method remains one of the most methodologically supported and conceptually coherent physiotherapeutic approaches for adolescents with idiopathic scoliosis.

## Figures and Tables

**Table 1:** Certainty of Evidence for Primary and Secondary Outcomes.

Outcome	Number of Studies	Study Quality	Consistency	Directness	Precision	Overall Certainty
Cobb angle improvement (Schroth only)	11	Moderate- High	High	Direct	Moderate	Moderate-High
Cobb angle improvement (Schroth + brace)	7	High	High	Direct	Moderate	High
Angle of trunk rotation (ATR)	10	Moderate	Moderate	Direct	Moderate	Moderate
Postural improvements	9	Moderate	Moderate-High	Direct	Moderate	Moderate-High
Respiratory outcomes	4	Moderate	Moderate	Direct	Low-Moderate	Moderate
Quality of Life (SRS-22)	6	High	High	Direct	Moderate	High

Certainty of evidence was assessed using a simplified GRADE framework. Certainty ranged from moderate to high for Cobb angle improvement and quality of life outcomes, and moderate for ATR and respiratory outcomes.

**Table 2:** Characteristics of included studies evaluating Schroth-only interventions in adolescents with idiopathic scoliosis.

No.	Author & Year	Study Design	Sample (*n*), Age	Risser	Curve Type/Baseline Cobb	Intervention Protocol	Main Outcomes
1.	Otman 2005 [7]	RCT	*n* = 50, age 10–17	0–3	Thoracic/TL; Cobb 20–40°	12 weeks Schroth, supervised 3×/week + home program	Cobb ↓; ATR ↓; posture improved
2.	Kuru 2016 [8]	RCT	*n* = 45, age 10–18	0–4	Thoracic/TL; Cobb 20–45°	months Schroth, 2 supervised sessions/week	Significant Cobb ↓ vs. control (*p* < 0.05)
3.	Kim 2016 [9]	RCT	*n* = 34, age 12–18	0–4	Mild AIS; Cobb 10–25°	Schroth vs. Pilates, 12 weeks	Better trunk symmetry & ATR ↓ with Schroth
4.	Hwangbo 2016 [10]	Controlled trial	*n* = 28, age 12–17	0–2	Cobb 15–30°	8-week Schroth program	Improved posture + psychological indices
5.	Kocaman 2021 [11]	RCT	*n* = 40, age 10–17	0–4	Cobb 20–45°	10-week Schroth vs. core stabilization	Schroth superior for Cobb ↓ & posture
6.	Aktan 2022 [12]	Prospective clinical	*n* = 32, age 10–18	0–3	Cobb 15–40°	Intensive camp, 5 days/week	Posture, ATR, balance improved
7.	Mohamed RA 2021 [13]	RCT	*n* = 36, age 12–17	0–4	Thoracolumbar; Cobb 20–40°	Schroth vs. PNF, 10 weeks	Both improved; Schroth better in ATR
8.	Mohamed A 2022 [14]	RCT	*n* = 30, age 11–17	0–4	Cobb 20–40°	Schroth ± hippotherapy; 12 weeks	Pulmonary gains > Schroth alone
9.	Büyükturan 2024 [15]	RCT	*n* = 56, age 10–18	0–4	Lenke 1; Cobb 20–45°	Schroth vs. Lyon, 12 weeks	Greater ATR & Cobb ↓ with Schroth
10.	Zhang 2024 [16]	RCT	*n* = 48, age 11–16	0–3	Cobb 15–35°	Schroth ± pelvic rotation correction	Additional pelvic correction > Schroth alone
11.	Kaya 2025 [17]	RCT	*n* = 44, age 10–18	0–4	Cobb 20–40°	Schroth vs. PNF, 8 weeks	Schroth superior in Cobb & symmetry

Note: AIS = adolescent idiopathic scoliosis; TL = thoracolumbar; ATR = angle of trunk rotation; PNF = proprioceptive neuromuscular facilitation; RCT = randomized controlled trial; ↑ = increase; ↓ = decrease.

**Table 3:** Characteristics of included studies evaluating Schroth exercises combined with bracing in adolescents with idiopathic scoliosis.

No.	Author & Year	Study Design	Sample (*n*), Age	Risser	Brace Type + Cobb	Schroth Protocol	Main Outcomes
1.	Schreiber 2015 [6]	RCT	*n=* 50, age 10–18	0–3	TLSO brace; Cobb 20–45°	6 months Schroth + brace	Better Cobb ↓, SRS-22 ↑ vs. brace alone
2.	Kwan 2017 [18]	Prospective controlled	*n* = 30, age 10–17	0–3	TLSO; Cobb 20–40°	6 months supervised Schroth	Reduced progression vs. brace alone
3.	Schreiber 2019 [19]	RCT	*n* = 38, age 10–18	0–3	TLSO; Cobb 20–45°	Schroth 3×/week + home	Patient-perceived improvement ↑
4.	Mohamed N 2024 [20]	Controlled	*n* = 40, age 10–17	0–3	TLSO; Cobb 15–40°	Schroth 12 weeks	3D surface topography improved
5.	Stein 2024 [21]	Controlled	*n* = 44, age 10–18	0–3	Brace; Cobb 20–40°	Intensive Schroth + brace	Respiratory mechanics ↑; function ↑
6.	Kyrkousis 2024 [22]	RCT	*n* = 50, age 11–17	0–3	Rigo/Chêneau; Cobb 20–45°	6 months Schroth + brace	Lower progression vs. brace alone

Note: TLSO = thoracolumbosacral orthosis; 3D = three-dimensional; RCT = randomized controlled trial.

**Table 4:** Risk of Bias Assessment for the Included Studies.

Study	Design	Randomization/Allocation	Deviations from Intended Interventions	Missing Data	Outcome Measurement	Selective Reporting	Overall Risk of Bias
Otman 2005 [7]	RCT	Some concerns	Low	Low	Some concerns	Low	Some concerns
Kuru 2016 [8]	RCT	Low	Low	Low	Low	Low	Low
Kim 2016 [9]	RCT	Some concerns	Low	Low	Some concerns	Low	Some concerns
Hwangbo 2016 [10]	Controlled	Moderate	Low	Low	Moderate	Low	Moderate
Kocaman 2021 [11]	RCT	Low	Low	Low	Low	Low	Low
Aktan 2022 [12]	Prospective	Moderate	Low	Low	Moderate	Low	Moderate
Mohamed RA 2021 [13]	RCT	Some concerns	Low	Low	Low	Low	Some concerns
Mohamed A 2022 [14]	RCT	Some concerns	Low	Low	Low	Low	Some concerns
Büyükturan 2024 [15]	RCT	Low	Low	Low	Low	Low	Low
Zhang 2024 [16]	RCT	Low	Low	Low	Low	Low	Low
Kaya 2025 [17]	RCT	Low	Low	Low	Low	Low	Low
Schreiber 2015 [6]	RCT	Low	Low	Low	Low	Low	Low
Kwan 2017 [18]	Controlled	Moderate	Low	Low	Low	Low	Moderate
Schreiber 2019 [19]	RCT	Low	Low	Low	Low	Low	Low
Mohamed N 2024 [20]	Controlled	Moderate	Low	Some concerns	Moderate	Some concerns	Moderate
Stein 2024 [21]	Controlled	Moderate	Low	Low	Moderate	Low	Moderate
Kyrkousis 2024 [22]	RCT	Low	Low	Low	Low	Low	Low

Abbreviations: RCT—randomized controlled trial. Risk of bias categories are reported according to the Cochrane RoB 2.0 tool.

## Data Availability

Data sharing is not applicable to this article as no new data were created or analyzed.

## Supplementary Materials

The following supporting information can be downloaded at: https://www.mdpi.com/article/10.3390/jcm15031266/s1, PRISMA 2020 Statement.

## Abbreviations

The following abbreviations are used in this manuscript:

AIS	Adolescent Idiopathic Scoliosis
ADL	Activities of daily living
AP	Anteroposterior
ATR	Angle of Trunk Rotation
AVR	Apical Vertebral Rotation
BOG	Brace Only Group
BSG	Brace + Schroth Group
CA	Cobb Angle
CG	Control Group
CI	Confidence Interval
FVC	Forced Vital Capacity
FEV1	Forced Expiratory Volume in 1 Second
GRC	Global Rating of Change
LG	Lyon Group
MVV	Maximum Voluntary Ventilation
n	Sample Size
PA	Posteroanterior
PEG	Pilates Exercise Group
PNF	Proprioceptive Neuromuscular Facilitation
PNF-G	Proprioceptive Neuromuscular Facilitation Group
PRISMA	Preferred Reporting Items for Systematic Reviews and Meta-Analyses
PSSE	Physiotherapeutic Scoliosis-Specific Exercises
QoL	Quality of Life
RCT	Randomized Controlled Trial
Risser	Risser Staging System
SEG	Schroth Exercise Group
SG	Schroth Group
SRS	Scoliosis Research Society
SRS-22	Scoliosis Research Society-22 Questionnaire
WRVAS	Walter Reed Visual Assessment Scale
X-ray	Radiography
